# Exploring Non-Expert Robot Programming Through Crowdsourcing

**DOI:** 10.3389/frobt.2021.646002

**Published:** 2021-07-29

**Authors:** Sanne van Waveren, Elizabeth J. Carter, Oscar Örnberg, Iolanda Leite

**Affiliations:** Robotics, Perception, and Learning, KTH Royal Institute of Technology, Stockholm, Sweden

**Keywords:** human-robot interaction, non-expert robot programming, crowdsourcing, block-based programming, robots

## Abstract

A longstanding barrier to deploying robots in the real world is the ongoing need to author robot behavior. Remote data collection–particularly crowdsourcing—is increasingly receiving interest. In this paper, we make the argument to scale robot programming to the crowd and present an initial investigation of the feasibility of this proposed method. Using an off-the-shelf visual programming interface, non-experts created simple robot programs for two typical robot tasks (navigation and pick-and-place). Each needed four subtasks with an increasing number of programming statements (if statement, while loop, variables) for successful completion of the programs. Initial findings of an online study (N = 279) indicate that non-experts, after minimal instruction, were able to create simple programs using an off-the-shelf visual programming interface. We discuss our findings and identify future avenues for this line of research.

## 1 Introduction

As robots move out of controlled industrial environments into the real world, a persistent challenge is the need to expand robot behavior to adapt and respond to real-world situations without constant expert supervision. As an example, consider a robot guide that is, developed to operate in a space where doors are always open; if it is placed in new surroundings where it encounters a closed door, it might encounter a failure case since it might not be programmed with the right sequence of actions that specify to open the door before proceeding. Versatility is crucial for robots to successfully complete a wide variety of tasks, and it relies greatly on the ease with which robots can be programmed (Lozano-Perez, 1983).

To date, robot programming has primarily been a task for engineers that required a high level of mathematical and programming knowledge. This has limited the amount of programs created and, in turn, versatility. One way to support versatile robots is to be able to monitor them and create simple robot programs on the fly, which could potentially include expanding the ability to program robots to non-experts. While short-horizon skill learning, such as pushing and grasping objects, has typically been taught through small-scale learning from demonstration (LfD) (Argall et al., 2009) or teleoperation-like control, higher-level behavior (the order of actions to accomplish a task successfully) or additional rules (move to the next room, but if the door is closed open the door first) could be scaled to the crowd and directly provided in the form of simple robot programs. The main advantage to provide such additional rules and high-level robot behaviors using crowdsourcing is the quick and easy access to a large pool of non-experts. As an example, in a pick-and-place task, if a robot’s task changes from picking up any item from a tray to picking up only pink items, a non-expert crowdworker anywhere on earth, at any time of the day, can provide a rule that specifies in which order and under which conditions actions should be executed (If item is pink → Pick up item). Many robot tasks or rules require knowledge that people commonly have, such as household tasks or interaction tasks. Using crowdworkers to monitor robots may be beneficial and efficient, as they can monitor a number of such scenarios at a time without the need to be on site.

However, this method of recruiting novices relies on people being able to understand how to program different robots quickly. Previous literature has used visual programming interfaces to lower the barrier of entry for novice programmers. For example, many researchers have developed dataflow-based visual programming environments to this end (Glas et al., 2012; Sauppé and Mutlu, 2014; Alexandrova et al., 2015; Glas et al., 2016; Porfirio et al., 2018; Suguitan and Hoffman, 2019). One limiting factor of dataflow-based interfaces is that they may not always scale well with large space states (Glas et al., 2016; Huang et al., 2016). This can make it difficult to modify or remove elements, as the diagram can quickly become a jumble of elements and their connections (Coronado et al., 2020).

Another potential method to allow novices to program is the use of block-based interfaces (e.g., Pot et al., 2009; Datta et al., 2012; Chung et al., 2016; Huang et al., 2016; Huang and Cakmak, 2017; Paramasivam et al., 2017; Coronado et al., 2018; Coronado et al., 2019). We are inspired by these works and interested in extending these methods to let non-expert crowdworkers provide additional rules and exceptions to typical robot behaviors. In this work, we focus on investigating if/how non-experts can create simple robot programs using an off-the-shelf visual interface without extensive training, rather than designing and developing a novel interface. Leveraging prior work that focused on the interface design, we are interested to see whether such off-the-shelf interfaces can be customized to fit our particular robot programming use case.

In addition to needing a method for users to program the robot, we must consider how best to leverage a remotely shared environment. Situated tangible programming has allowed users with little-to-no training to program robots while situated in the task context by using programming blocks to reference objects (Sefidgar et al., 2017; Sefidgar and Cakmak, 2018). An extension of this method included real-time feedback projected onto the robot’s workspace and was used effectively by people with prior traditional programming experience (Sefidgar et al., 2018). Stenmark et al. (2017) developed a graphical interface with built-in basic actions, tested with users with some technical or mathematical experience, to create new robot skills. Unlike these works, we explore collecting additional rules for a robot rather than the generation of new skills and focus our attention on off-site non-expert users.

Another consideration for our remote, novice programming proposal is whether this work can be crowdsourced effectively. The success of crowdsourcing in other tasks has been shown extensively in prior work, including for spoken dialog generation for conversational systems (Lasecki et al., 2013; Mitchell et al., 2014; Leite et al., 2016; Yu et al., 2016; Guo et al., 2017; Kennedy et al., 2017; Huang et al., 2018; Jonell et al., 2019) and interaction data and non-verbal behavior (Orkin and Roy, 2007; Orkin and Roy, 2009; Chernova et al., 2010; Rossen and Lok, 2012; Breazeal et al., 2013; Sung et al., 2016). In previous research that is, more relevant to ours, Lee and Ko (2011) crowdsourced non-expert programmers for an online study and found that personified feedback of a robot blaming itself for errors increased the non-programmers’ motivation to program. While this work demonstrated promise for using the crowd for program correction, we focus instead on program creation by non-expert crowdworkers.

In this paper, we created an initial experiment that used block-based programming for crowdworkers to see how well they could create simple robot programs on a virtual robot. It is important to recognize that our aim is to investigate if/how people can create simple robot programs, and this work is a first step towards non-expert robot programming. As discussed in Section 5, future avenues of work should address the transfer between our simulation environment and real world environments. There are numerous use cases in which crowdsourcing robot programs could be promising. For example, a mobile robot in a nursing home assisting human workers could use help to check whether all residents have left their rooms right before meal time. Also, a mobile robot that guides people to their gate at the airport could be instructed to consider taking a longer, but more suitable route (e.g., without stairs) in special cases, such as when guiding elderly or visually impaired people.

As a first step, we showed that non-expert crowd workers can program robots on two typical robot tasks after up to 1.5 min of instructions. However, workers seemed to experience difficulty with correct use of loop blocks, block placement, and variable use. Exploring people’s common mistakes and commentary after they completed the task, we discuss future directions of non-expert robot programming through crowdsourcing. This paper provides the following contributions:

1. A proposed method for scalable non-expert robot programming through crowdsourcing;
2. An exploration of the extent to which non-expert crowd workers can successfully create robot programs using an off-the-shelf visual programming interface after minimal training through an analysis of commonly made mistakes from non-experts crowd workers’ created programs.

## 2 Related Work

This work aims to contribute to new ways of large-scale robot programming for real-world applications. As pointed out by Leite et al. (2013), a long-standing barrier to deploying robots in the real-world is the ongoing need to author robot behavior. For robots to be deployed in naturalistic environments without constantly breaking down and requiring expert assistance, they need to be able to constantly add new behavior (sequences) to their existing repertoire. Typically, engineers and robot programmers have been responsible for authoring robot behaviors, an approach that does not scale well to real-world, long-term interactions (Leite et al., 2016). Recently, several studies have explored ways to make robot programming more accessible to people with varying levels of programming expertise.

We need to recognize that programming can be intimidating for novices (Eisenberg, 1997), due to high cognitive load, complex languages, and error-proneness (Bau et al., 2017). Drag-and-drop graphical interfaces, such as Google’s Blockly and MIT’s Scratch, have been commonly used to facilitate this process. In these interfaces, visual blocks represent programming procedures which can be visually snapped together, eliminating the need to write actual code and deal with syntax errors (Trower and Gray, 2015). Block-based languages focus on recognition of blocks instead of recalling programming vocabulary, reduce cognitive load by chunking code into meaningful blocks, and reduce error-proneness as users do not need to write code (Bau et al., 2017).

Many researchers have developed flow-based visual programming environments for robot programming (Sauppé and Mutlu, 2014; Alexandrova et al., 2015; Glas et al., 2016; Suguitan and Hoffman, 2019), but these interfaces may not always scale well with large space states (Glas et al., 2016; Huang et al., 2016). Systems such as Blossom (Suguitan and Hoffman, 2019), Interaction Blocks (Sauppé and Mutlu, 2014), and RoboFlow (Alexandrova et al., 2015) allow users to specify interaction patterns or behavior sequences for social robots. Alternatively, other works have explored the use of visual drag-and-drop interfaces (Pot et al., 2009; Datta et al., 2012; Chung et al., 2016; Huang et al., 2016; Huang and Cakmak, 2017; Paramasivam et al., 2017; Coronado et al., 2018; Coronado et al., 2019). One series of these includes CustomPrograms (Huang et al., 2016), iCustomPrograms (Chung et al., 2016), and Code3 (Huang and Cakmak, 2017), all of which allowed non-experts to program a robot to perform basic social behaviors with minimal training. Code3 lets users create models of objects to use to teach the robot new behaviors through demonstration and program the robot through a visual programming interface called CodeIt (Paramasivam et al., 2017).

Situated tangible programming has allowed users with minimal training to program robots while situated in the task context, using programming blocks to reference objects (Sefidgar et al., 2017). An extension of this method included real-time feedback projected onto the robot’s workspace and was used effectively by people with prior traditional programming experience (Sefidgar et al., 2018).

Stenmark et al. (2017) developed a graphical interface with built-in basic actions, tested with users with some technical or mathematical experience, to create new robot skills. Unlike this work, we focus on collecting robot behavior from predefined actions rather than the generation of new skills.

Prior work has used crowdsourcing for spoken dialog generation for conversational systems (Jurčíček et al., 2011; Lasecki et al., 2013; Mitchell et al., 2014; Leite et al., 2016; Yu et al., 2016; Guo et al., 2017; Kennedy et al., 2017; Huang et al., 2018; Jonell et al., 2019), interaction data and non-verbal behavior (Orkin and Roy, 2007, Orkin and Roy, 2009; Chernova et al., 2010; Rossen and Lok, 2012; Breazeal et al., 2013; Sung et al., 2016). However, no previous work created a method to collect new robot behaviors for day-to-day tasks on a large scale using semi-situated non-experts.

In sum, non-expert robot programming and crowdsourcing have both been explored in previous robotics and human-robot interaction (HRI) research. To date, no research has combined these two approaches to examine the opportunities and challenges of large-scale remote non-expert robot programming as a method for robot behavior collection.

## 3 Materials and Methods

### 3.1 Robot Programming Interface

We built a visual programming interface based on MIT’s Scratch (Resnick et al., 2009), an intuitive, accessible drag-and-drop programming tool using a general-purpose language for which prior work has shown effectiveness for non-experts (Malan and Leitner, 2007). As previously discussed, visual programming is a popular tool for non-expert programming, and it particularly fits our robot programming through crowdsourcing use case as it allows for intuitive, non-expert robot programming without the need for extensive training.

**FIGURE 1:**
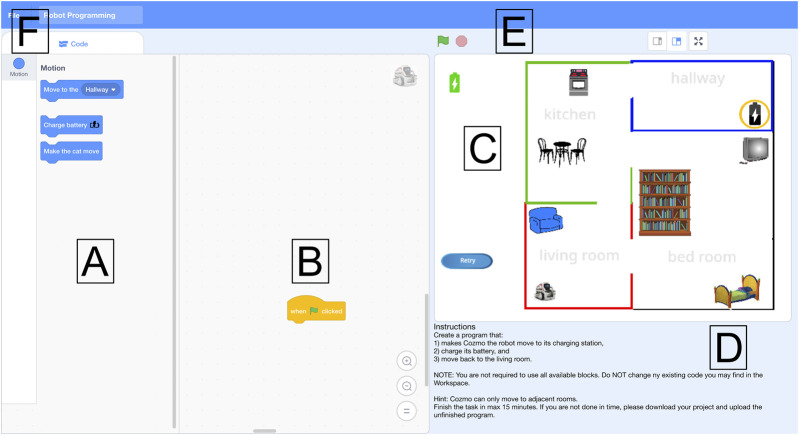
The interface consists of **(A)** blocks to drag and drop to the **(B)** workspace. The **(D)** instructions explain the task. The program can be **(E)** run and stopped, and the program’s output is visualized in the **(C)** stage. Finally, users can **(F)** download their program.

We adapted the original Scratch interface to provide our workers with a clean interface tailored to the current robot programming task (see Figure 1). The interface provides the user with predefined code blocks that we can flexibly define for each task. We defined a set of blocks to test the desired programming statements; blocks and functionality irrelevant to the task were removed. The blocks can be dragged into the workspace and snapped together to form a program that produces the desired robot behavior. The program can be tested by clicking the green flag icon, and the program execution can be stopped by clicking the red stop button. When a program is tested, the stage (see Figure 1C) visualizes the program output. For any incorrect program, simple error messages state what is incorrect and encourage the user to retry. For the pick-and-place task, we created a 3D animated robot that moved its arms to illustrate picking and placing cylinders. The web-based application runs on most standard web browsers.

Workers on the crowdsourcing platform Amazon Mechanical Turk (AMT) were introduced to the problem through our Human Intelligence Task (HIT) that contained the instructions, the link to the web application with our visual programming interface, a questionnaire, and a file upload. When the web application was open in the worker’s web browser, the session, including cursor movement and mouse clicks, was screen-recorded and saved for qualitative analysis. Workers were requested to download their program and then upload it to our server to pass the HIT. This gave us the opportunity to analyze both the continuous process of the workers’ programming and the finished program.

### 3.2 Robot Programming Tasks

**FIGURE 2:**
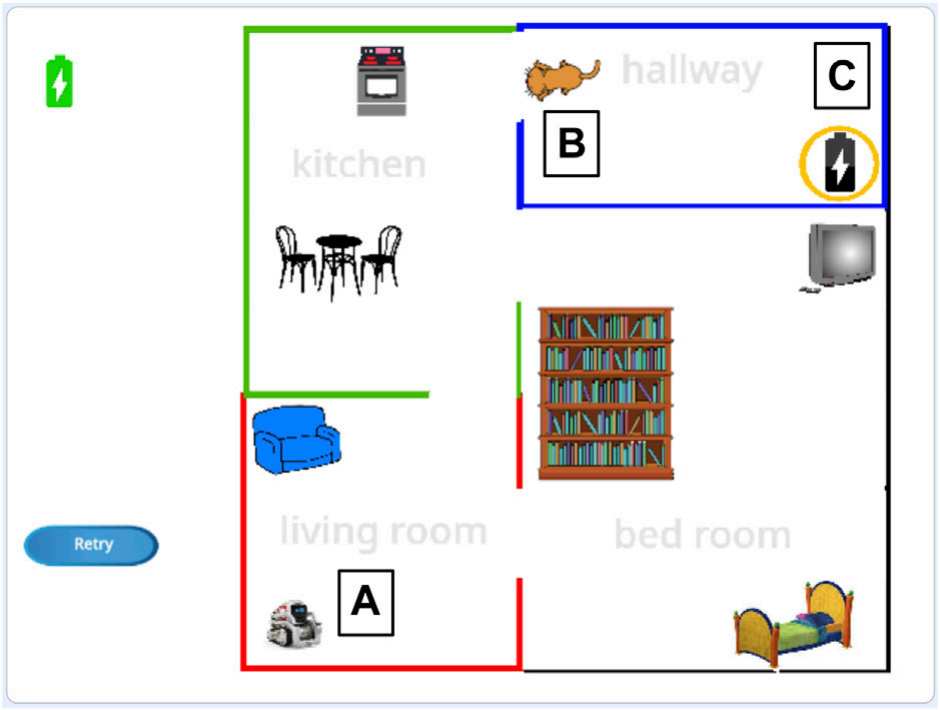
The navigation task in which the robot **(A)** needs to move from the living room, through the kitchen, to the hallway and to the charging station **(C)**, charge its battery, and move back. If the cat **(B)** (controlled by a variable that is either or 1) blocks the door, the robot first needs to make the cat move.

**FIGURE 3:**
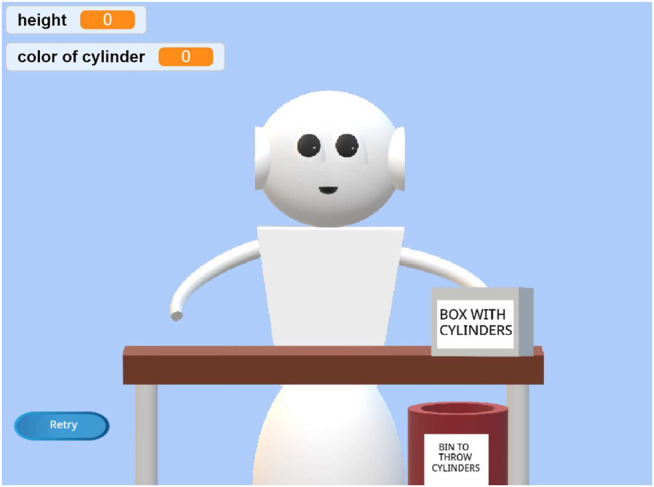
The pick-and-place task, in which a virtual robot needs to pick cylinders from a box on the table and place them correctly. If the chosen cylinder’s color is pink, the robot must place it on the table; otherwise it must be put in the bin below the table. It should stack cylinders until the stack’s height is 3.

The two tasks are informed by prior research on robot programming. One task is a navigation task (see Figure 2) in which the robot needs to move to predefined locations, inspired by Huang et al. (2016). The other task is a pick-and-place task (see Figure 3), in which the robot needs to stack cylinders, similar to Sefidgar et al. (2017). These are typical tasks for which it is likely that a robot would need to adapt its behavior based on the particular environment in which it is deployed (e.g., different factory, home, etc.). However, as mentioned earlier, our crowdsourced robot programming use case is illustrated by but not limited to these two tasks.

The interface contains program blocks and program statements; program blocks can contain other program blocks and statements, and a program statement can contain program variables. The tasks are designed such that different programming statements (blocks, conditionals, loops, and variables) can be gradually introduced, which allows for flexible definition of new programming statements. We are interested to see for what combinations of programming statements non-experts can successfully create programs, so we introduced an increasing number of programming statements rather than comparing programming statements individually. We created four subtasks for each task to explore at which levels difficulties would arise:

• Blocks subtask: in which the robot can be programmed with some simple blocks that are formed from simple control structures; this subtask only involved dragging and dropping blocks (e.g., “move to room,” where the room could be chosen from a drop-down menu);
• Conditional subtask: that uses a decision statement introduced in the form of an if-else conditional;
• Loop subtask: that includes the if-else conditional from the conditional subtask and additionally introduces an iteration statement in the form of a loop (e.g., repeat until a certain condition is satisfied, similar to a while-loop);
• Variables subtask: that includes both an if-else conditional and a loop, and also introduces variables and operators that need to be used to specify the conditions that must be satisfied for the conditional and loop to be triggered.

To fit the typical duration of HITs, which are usually short tasks, the instructions of the tasks were kept to a minimum. In a pilot study (N = 3, one male, 2 female; μ age = 35.0, SD = 17.32), we had non-programmers talk aloud while they programmed a virtual robot in the navigation task, starting with no instructions, and providing increasingly more instructions. These pilots informed us that an abstract overview of the interface and its components were the minimally sufficient instructions. This is specifically desirable when creating programs for a wide range of situations for which one general instruction video can suffice. Prior to the task, participants watched a minute-long instruction video showing the basic usage of the interface. To gain some initial insight on the impact of instructions on task success, we ran an additional study in which people watched the same instruction video with an additional 30 s of instruction that illustrated the use of the loop programming statement. The 1-min and 1.5-min instruction videos can be found here, respectively: vimeo.com/492089177 and vimeo.com/492088923.

The task instructions were kept the same for each subtask, but the variables subtask included one sentence about the variables; e.g., in the navigation task: “When the cat is present the cat_present variable is 1 and otherwise 0. The fully_charged variable goes from 0 (empty battery) to 1 (fully charged).” For all subtasks, the maximum duration of the task was 15 min, and the maximum HIT time was 30 min.

#### 3.2.1 Navigation Task

**FIGURE 4:**
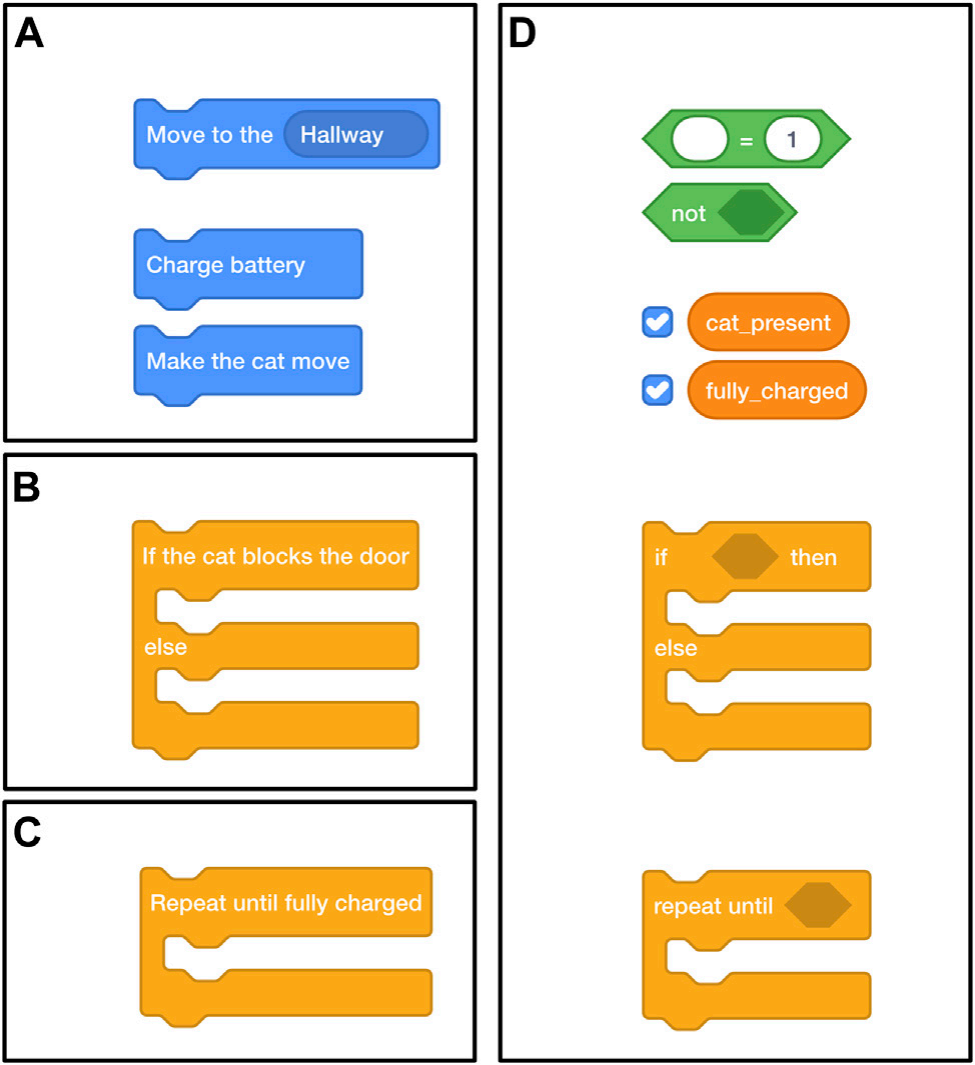
The blocks used in the navigation task, illustrating the blocks used in the different programming statements subtasks: **(A)** block statements only, **(B)** a conditional, **(C)** a loop, and a conditional and loop that require **(D)** variables.

In the blocks subtask of the navigation task, workers made a program using the available blocks (see Figure 4) to make the robot [located at (1) in Figure 2]: 1) move to its charging station located in the hallway; 2) charge its battery; and 3) move back to the living room. The robot could only move between rooms that were adjacent to each other and its battery would drain while it operated. In the conditional subtask, we introduced a cat [located at (2) in Figure 2] that would randomly appear and block the door from the kitchen to the hallway. This was not mentioned in the instructions. In the real world, sudden changes in the environment may cause robots to fail at a task. For example, the robot might get stuck when the cat blocks its way. This could also be a closed door, as mentioned as an example in the Introduction. In this case, workers could use a conditional to specify a sequence of actions that the robot should perform to continue. Here, they can specify that the robot should “make the cat move” if the cat blocked the door [see (2) in Figure 4]. In the loop subtask, the robot had to charge “until fully charged,” and we introduced a loop block [see (3) in Figure 4] that would repeat any code placed inside it until the condition was met. Finally, in the variable subtask, both the conditional and the loop stayed the same, but variables had to be used to specify the condition for these blocks; e.g., the “repeat until fully charged” changed to “repeat until placeholder,” where a variable could be placed inside the placeholder [see Figure 4(4)].

#### 3.2.2 Pick-and-Place Task

In the blocks subtask of the pick-and-place task, we asked people to create a program to make the robot pick up the green cylinder (out of three colored cylinders on the table) and place it on the pink one, then pick up the blue cylinder and put it on the green one. In the conditional subtask, the robot had to pick one cylinder from a box on the table and place it on the table if the color of the cylinder was pink or put it in a box under the table if it was not (see Figure 3). For this subtask, we introduced a conditional that would check if the picked cylinder was pink. The box contained three pink cylinders, a blue cylinder, and a green cylinder. The robot would pick up blocks in a random order so that users could not use the order to create a correct program. In the loop subtask, the robot had to stack cylinders if the cylinder’s color was pink until the stack’s height was three. This could be done using the loop block. In the variables subtask, the conditional and loop statements required variables inside an operator to specify what condition must be met to trigger the statements.

In both tasks, the robot may fail in its task, but in the navigation task it is an unexpected event that causes the robot to fail (a cat may randomly appear) inherent to the environment, whereas in the pick-and-place task it is embedded in the task instructions (only place a cylinder if the color is pink).

### 3.3 Experimental Method

In order to investigate the research questions outlined earlier, we conducted an online study using the system described in the previous section. We focused on program creation, similar to Sefidgar et al. (2017), as we were interested in the success rate for program creation by non-experts and what difficulties arose during program creation using an off-the-shelf interface. We collected data through crowdsourcing, giving us substantially more data to analyze for patterns of common challenges.

#### 3.3.1 Measures

From the task screen recordings, the collected programs and survey responses, we extracted the following measures.

##### 3.3.1.1 Task Success

From task recordings and uploaded programs, we counted how many people successfully completed each subtask. This means that the program did what was asked without errors.

##### 3.3.1.2 Task Duration

We manually extracted task duration as the number of seconds it took people to create their final program, regardless of task success. Due to some software issues with the external recording tool, we had N = 24 recordings for the blocks subtask, N = 24 for the conditional subtask, N = 17 for the loop subtask, and N = 23 for the variables subtask for the navigation task. For the pick-and-place task, we obtained N = 20 recordings for the blocks subtask, N = 19 for the conditional subtask, N = 22 for the loop subtask, and N = 20 for the variables subtask. We asked people to spend a maximum of 15 min on the task, after which they should submit their program regardless of whether it was correct. Naturally, we expected an increase in task duration with the increase in programming statements.

##### 3.3.1.3 Task Usability

To assess task usability, we adapted the widely used System Usability Scale (Brooke, 1996). A reliability analysis showed that the scale had acceptable reliability, α = 0.88, so we used the mean score in our analyses. We included the following items (R indicates reverse-scoring):

1. I think that I would like to do this task frequently as a HIT.
2. I found the programming task unnecessarily complex (R).
3. I thought the programming task was easy to perform.
4. I think that I would need the support of a technical person to be able to perform the programming task (R).
5. I would imagine that most people would learn to do this programming task very quickly.
6. I felt very confident doing the programming task.
7. I needed to learn a lot of things before I could get going with the programming task (R).
8. I found the programming task very challenging to perform (R).

##### 3.3.1.4 Task Load

We adapted the NASA Task Load Index (Hart and Staveland, 1988), removing the physical demand item and the original pair-wise comparisons as they were not relevant to the current task. The remaining five items, rated using a slider from 0 (labeled very low for item 1, 2, 4, and 5, and perfect for item 3) to 100 (labeled very high for item 1, 2, 4, and 5, and failure for item 3), were as follows:

1. Mental demand: How mentally demanding was the task?
2. Temporal demand: How hurried or rushed was the pace of the task?
3. Performance: How successful were you in accomplishing what you were asked to do?
4. Effort: How hard did you have to work to accomplish your level of performance?
5. Frustration: How insecure, discouraged, irritated, stressed and annoyed were you during the task?

A reliability analysis showed moderate scale reliability, α = 0.70, which could be increased to acceptable reliability, α = 0.80, if item 3 was deleted. Hence, we excluded performance and averaged the four remaining items to get the task load score.

##### 3.3.1.5 Open Question

To gain a more in-depth understanding of what difficulties people experienced during the task, we added the following open-ended question: “During the task, what did you find difficult? Did you have any problems?” Participants could write an open answer to this question in a text box.

#### 3.3.2 Participants

We recruited 344 participants online through AMT, of whom 65 participants were excluded due to missing data or non-serious attempts, resulting in a total of 279 participants. Of these, 105 participants completed the navigation task (71 male, 32 female; μ age = 34.21, SD = 9.28) with the original instructions and 84 participants (55 male, 27 female, 2 other; μ age = 32.34, SD = 8.77) with an additional 30 s of instructions. 90 participants did the pick-and-place task with the original instructions (58 male, 32 female; μ age = 34.8, SD = 11.33). To avoid learning effects that could influence our comparisons, participants could only perform one HIT. Participants were compensated 4 USD.

Of the 105 participants who completed the navigation task with 1-m instructions, 29 participants did the blocks subtask (20 male, 8 female, 1 other; μ age = 38.48, SD = 8.91), 29 did the conditional subtask (21 male, 7 female, 1 other; *μ* age = 35.52, *SD* = 9.94), 19 did the loop subtask (16 male, 3 female; *μ* age = 29.11, *SD* = 5.45), and 28 did the variables subtask (14 male, 14 female; *μ* age = 31.89, *SD* = 9.03).

Of the 84 participants who completed the navigation task with 1.5-m instructions, 29 (20 male, 8 female, 1 other; *μ* age = 29.24, *SD* = 7.53) performed the conditional subtask, 28 did the loop subtask (17 male, 11 female; *μ* age = 32.32, *SD* = 7.82), and 27 did the variables subtask (18 male, 8 female, 1 other; *μ* age = 35.37, *SD* = 10.03) of the navigation task.

For the 90 participants completed the pick-and-place task, 20 people did the blocks subtask (13 male, 7 female; *μ* age = 38.10, *SD* = 13.11), 22 did the conditional subtask (18 male, 4 female; *μ* age = 29.27, *SD* = 6.26), 23 did the loop subtask (16 male, 7 female; *μ* age = 34.17, *SD* = 10.54), and 25 did the variables subtask (11 male, 14 female; *μ* age = 37.60, *SD* = 12.57).

##### 3.3.2.1 Programming Experience

Programming experience was measured on four items adapted from prior work (Feigenspan et al., 2012), rated on a scale from 1 (very inexperienced) to 10 (very experienced). Reliability was high, *α* = 0.95; so we used the mean score. All people reported themselves as inexperienced at programming (navigation task: *μ* = 3.15, *SD* = 2.58, median = 1.5; navigation task with additional instructions: *μ* = 2.76, *SD* = 2.36, median = 1.63; pick-and-place task: *μ* = 3.81, *SD* = 2.74, median = 2.63), and on average had less than 2 years of overall programming experience (navigation task: *μ* = 1.70, *SD* = 2.81; navigation with additional instruction: *μ* = 2.33, *SD* = 4.68; pick-and-place task: *μ* = 2.22, *SD* = 4.23) and of professional experience (navigation task: *μ* = 0.89, *SD* = 1.87; navigation with additional instruction: *μ* = 0.82, *SD* = 2.64; pick-and-place task: *μ* = 1.38, *SD* = 2.49). A logistic regression showed no significant association between programming experience and task success, *p* = 0.37.

#### 3.3.3 Data Analysis Plan

We treated the four subtasks with different numbers of programming statements as independent variables. We analyzed success rates for each task and its subtasks. To test whether semi-situated robot programming could be done in a sensible amount of time with reasonable task load, we examined task duration for people who completed the task. We compared usability and task load for both success and failure cases.

We expected task duration and task load to increase and usability to decrease with the number of programming statements because of the increasing complexity of the programs. To see at which programming statement the complexity caused a significant difference in the aforementioned measures, we performed three pair-wise comparisons: the basic subtask with the conditional subtask, the conditional subtask with the loop subtask, and the loop subtask with the variables subtask, with Bonferroni corrections for multiple comparisons using *α* = 0.016 as the significance threshold.

To explore what difficulties people faced during program creation, we analyzed the task recordings and classified the types of errors for people who failed to successfully create a program. Additionally, two authors double-coded the answers to the open-ended question described in Section 3.3.1 to classify what challenges people reported. The answers were coded into one or more of the following categories: No problems, Task order: challenges related to figuring out the steps of the task, Blocks: challenges related to figuring out how the blocks work, Anticipation: challenges anticipating the robot’s environment, Interface: challenges related to the interface, and Other. Fuzzy κ (Kirilenko and Stepchenkova, 2016) showed acceptable inter-rater agreement, κ = 0.7.

To study the impact of instructions on performance, we compared rates of task success in the conditional, loop, and variables subtasks of the navigation task across both instruction levels. We did not run any other tests to compare the tasks due to differing task designs.

## 4 Results

### 4.1 Navigation Task

#### 4.1.1 Task Success

**FIGURE 5:**
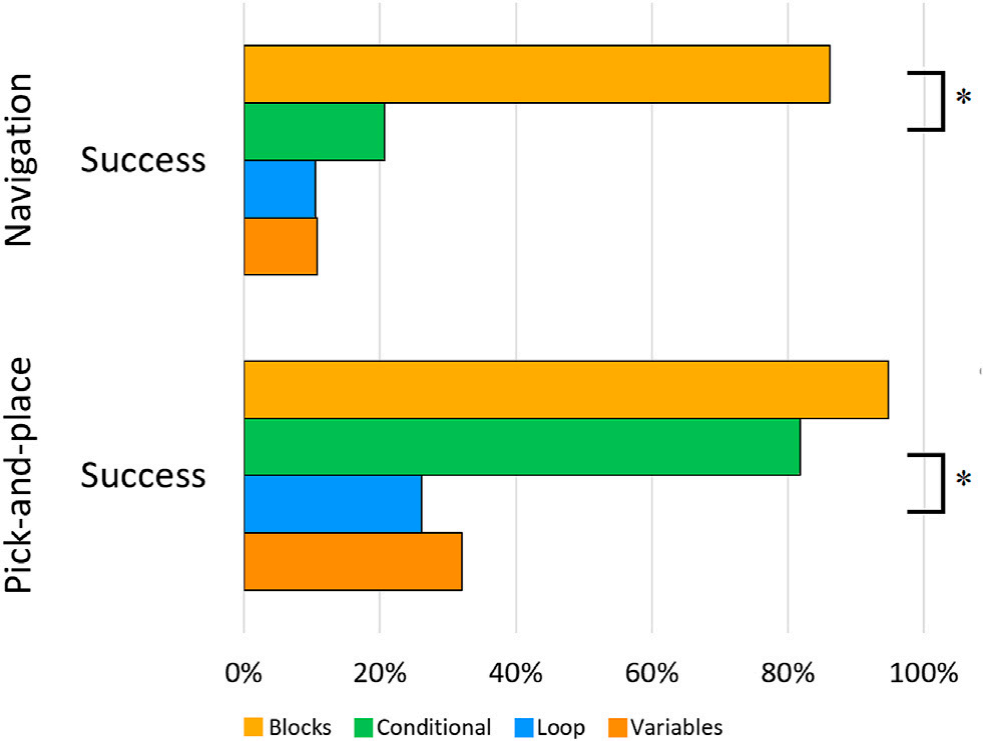
Percentage of successfully completed programs per subtask per task. * indicates significant differences (*p* < 0.016) (Recall that each task is only compared statistically to the task directly below it, for a total of three pairwise comparisons).

Overall, 34% of participants successfully completed the original task. Most of them finished the blocks subtask (86.2% success rate) rather than the conditional (20.7%), loop (10.5%), or variables subtasks (10.7%) (see Figure 5). A Chi-square test of independence found a significant relationship between programming concepts and task success, *χ2* (3, *N* = 105) = 48.32, *p* < 0.0001. Specifically, people who created programs that required a conditional statement (20.7%) were less likely to successfully complete the task than people in the blocks subtask (86.2%), *χ(1)* = 25.02, *p* < 0.0001. There were no significant differences between the conditional and loop subtasks or the loop and variables subtasks. For the navigation task with 1.5-min instructions, the task success was 33.3% for the conditional subtask, 28.6% for the loop subtask, and 7.7% for the variables task. Task success did not differ significantly between this version of the navigation task and the one with 1-min instructions, ps > 0.1.

#### 4.1.2 Task Duration

Across all subtasks, the mean task duration for successful tasks was 349 s (s) (SD = 278 s). The task duration appeared to increase with the number of programming statements: starting with the blocks subtask (*μ* = 250.48 s, *SD* = 217.98, median = 190.0), then the conditional subtask (*μ* = 390.0 s, *SD* = 211.82, median = 365.0), followed by the loop subtask (*μ* = 503.50 s, *SD* = 443.35, median = 503.50), and finally, the variables subtask (*μ* = 690.0 s, *SD* = 256.32, median = 720.0). However, Mann-Whitney U tests for the task duration in seconds showed no significant differences between the subtasks for people who successfully completed the program, ps > 0.07.

#### 4.1.3 Task Usability

We observed a decrease in task usability with the increase in programming concepts, with the blocks subtask having the highest average task usability score (*μ* = 4.47, *SD* = 0.74, median = 4.88), followed by the conditional subtask (*μ* = 4.16, *SD* = 0.70, median = 4.13), the loop subtask (*μ* = 3.95, *SD* = 0.65, median = 3.88), and, finally, the variables subtask (*μ* = 3.13, *SD* = 0.87, median = 3.13).

Mann-Whitney *U* tests of independent samples were used to examine the relationship between the task usability score and the different subtasks. Results revealed a statistically significant difference between the blocks subtask and the conditional subtask, *U* = 263.50, *Z* = −2.46, *p* = 0.014. Specifically, people in the blocks subtask rated the task usability higher (mean rank = 34.91) than did the people who did the conditional subtask (mean rank = 24.09). There was also a significant difference between the loop subtask and the variables subtask, *U* = 118.50, *Z* = −3.21, *p* = 0.001, where people who did the loop subtask rated usability higher (mean rank = 31.76) than those who did the variables subtask (mean rank = 18.73).

#### 4.1.4 Task Load

Task load appeared to increase with the increasing number of concepts, with the lowest average task load for the blocks subtask (*μ* = 29.71, *SD* = 20.55, median = 24.50), followed by the conditional subtask (*μ* = 40.16, *SD* = 24.79, median = 44.0) and the loop subtask (*μ* = 42.01, *SD* = 17.30, median = 34.50), which have similar means to each other, and, finally, the variables subtask (*μ* = 62.86, *SD* = 16.23, median = 60.88). A Mann-Whitney *U* test showed a statistically significant difference between the loop subtask (mean rank = 15.39) and the variables subtask (mean rank = 29.84), *U* = 102.50, *Z* = −3.55, *p* < 0.0001.

#### 4.1.5 Common Challenges

**FIGURE 6:**
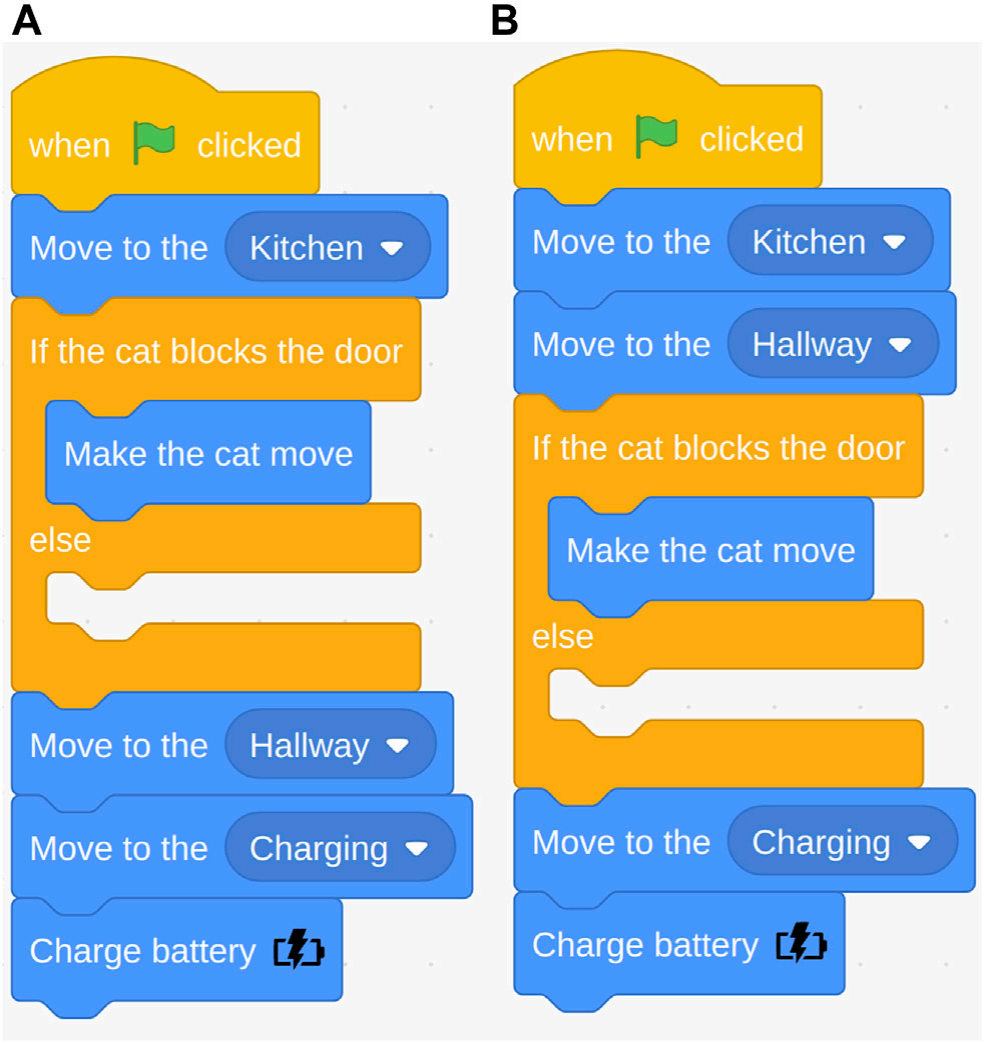
Navigation task: Correct program **(A)** and improper order of robot action (move) and if-else conditional **(B)**.

**FIGURE 7:**
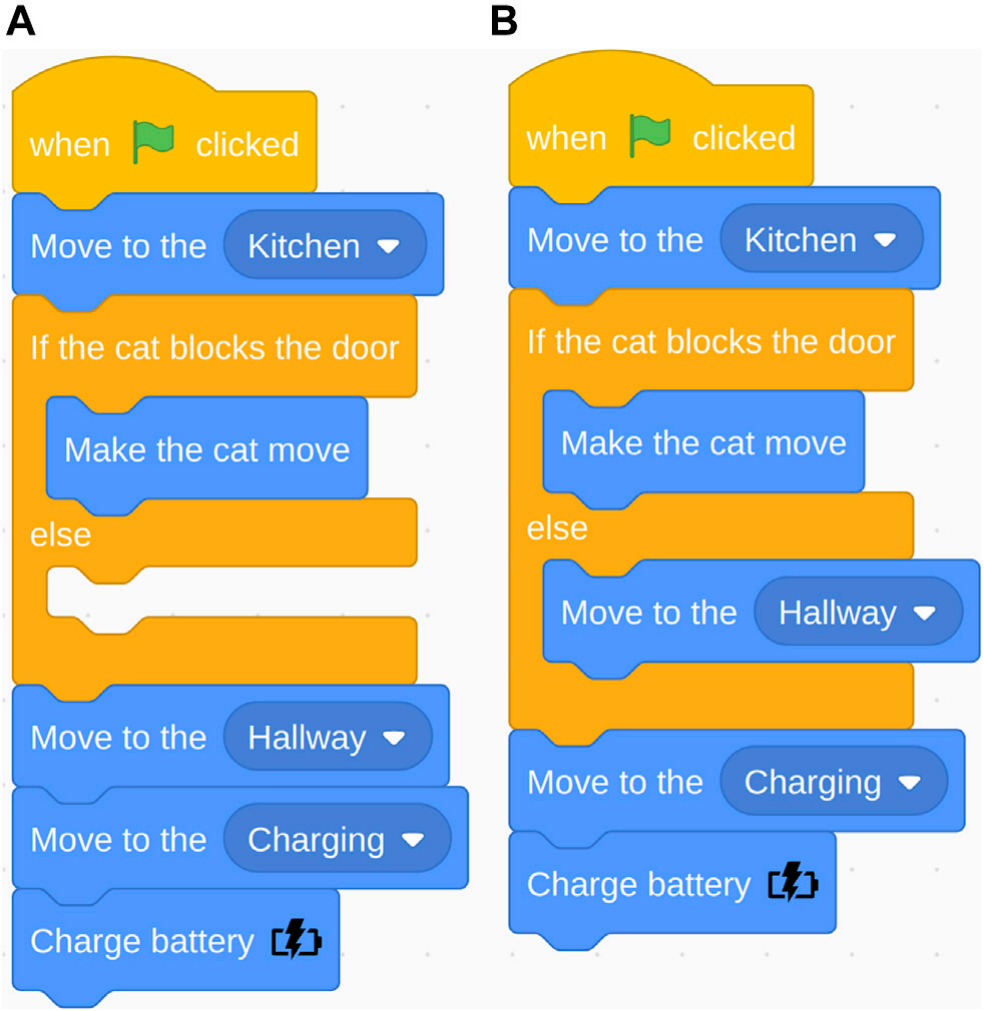
Navigation task: Correct program **(A)** and the “else” in the if-else conditional incorrectly used as “then” **(B)**.

We manually classified improper use and no use of the three programming statements (e.g., conditional, loop, and variables (see Table 1)). The most common error was the order of blocks related to the conditional statement. Many participants placed the “move to hallway” block before the conditional statement (see Figure 6). The robot first needed to check if the cat was blocking the door before it can proceed to the hallway; however, many people placed the conditional check after “move to hallway.” For example, one participant mentioned “(A problem was) grasping (…) if I should make sure the cat was out of the way before going into the Hallway or the inverse (…)” (loop subtask). Another participant said: (…) It was unclear to me if (…) we (make) the cat move to ENTER the hall (thus it should happen in the kitchen), or making the cat move as we enter the first bit of hallway?” (variables subtask). The second most common error was improper use of the conditional statement. The “else” in the conditional was treated as a “then” (see Figure 7). Consequently, the action placed in the “else” would only be executed if the condition was not met, but it should always execute. 23% of the participants’ open answers were coded as issues with Anticipation of the environment, e.g., when the cat would appear and the robot’s battery life.

**TABLE 1:** Classification of mistakes in the navigation task (improper use, IU; no use, NU) per programming statement per subtask [conditional (C), loop (L), and variables (V)].

Subtask	If-else		Loop		Variables	
	IU	NU	IU	NU	IU	NU
C	19 (68%)	2 (7%)	N/A	N/A	N/A	N/A
L	13 (72%)	2 (11%)	4 (22%)	1 (6%)	N/A	N/A
V	12 (44%)	9 (33%)	3 (11%)	9 (33%)	6 (22%)	11 (41%)

### 4.2 Pick-and-Place Task

#### 4.2.1 Task Success

Roughly 56% of all participants successfully completed the task, of whom most completed the blocks subtask (94.7% success rate) or the conditional subtask (81.8%), whereas the loop and variables subtask had 26.1 and 32.0% success rates, respectively (see Figure 5). A *χ2* test of independence found a significant relationship between programming concepts and task success, *χ2* (3, *N* = 89) = 31.75, *p* < 0.0001. People who created programs in the conditional subtask were more likely (81.8%, mean rank = 29.41) to successfully complete the program than people in the loop subtask (26.1%), *χ(1)* = 14.03, *p* < 0.0001.

#### 4.2.2 Task Duration

Again, we expected the task duration to increase with the number of programming concepts; therefore, we performed the planned pairwise comparisons. For the pick-and-place task (across all subtasks), the mean task duration for successful tasks was 3 min and 44 s (*SD* = 2 min and 44 s). For successful cases, the task duration (in seconds) appeared to increase with the number of programming statements, but the task duration in the conditional subtask (*μ* = 128.38, *SD* = 81.26, median = 117.0) appeared lower than in the blocks subtask (*μ* = 194.28, *SD* = 83.94, median = 172.0), followed by the loop subtask (level 3, *μ* = 303.0, *SD* = 281.57, median = 195.5), and finally, the variables subtask (*μ* = 355.5, *SD* = 110.35, median = 373.0). Mann-Whitney *U* tests for the task duration in seconds showed no significant differences in task duration for people who successfully completed the program between the subtasks, ps > 0.018.

#### 4.2.3 Task Usability

Task usability decreased with the increase in programming concepts, with the blocks subtask having the highest average usability score (*μ* = 4.41, *SD* = 0.65, median = 4.69), followed by the conditional subtask (3.83, *SD* = 0.90, median = 3.88), the loop subtask (*μ* = 3.63, *SD* = 0.95, median = 3.50), and finally, the variables subtask (*μ* = 3.46, *SD* = 1.13, median = 4.0). However, Mann-Whitney *U* tests of independent samples showed no statistically significant differences between subtasks, ps > 0.03.

#### 4.2.4 Task Load

Task load was lowest for the blocks subtask (*μ* = 28.96, *SD* = 17.10, median = 32.75). The task loads for the conditional (*μ* = 45, *SD* = 18.66, median = 42.0), the loop (*μ* = 51.93, *SD* = 21.21, median = 57.50) and variables subtasks (*μ* = 49.51, *SD* = 23.08, median = 43.50) were similar to each other. A Mann-Whitney U test showed only a statistically significant difference between the blocks subtask (mean rank = 16.68) and the conditional subtask (mean rank = 25.89), *U* = 123.50, *Z* = −2.43, *p* = 0.014.

#### 4.2.5 Common Challenges

**FIGURE 8:**
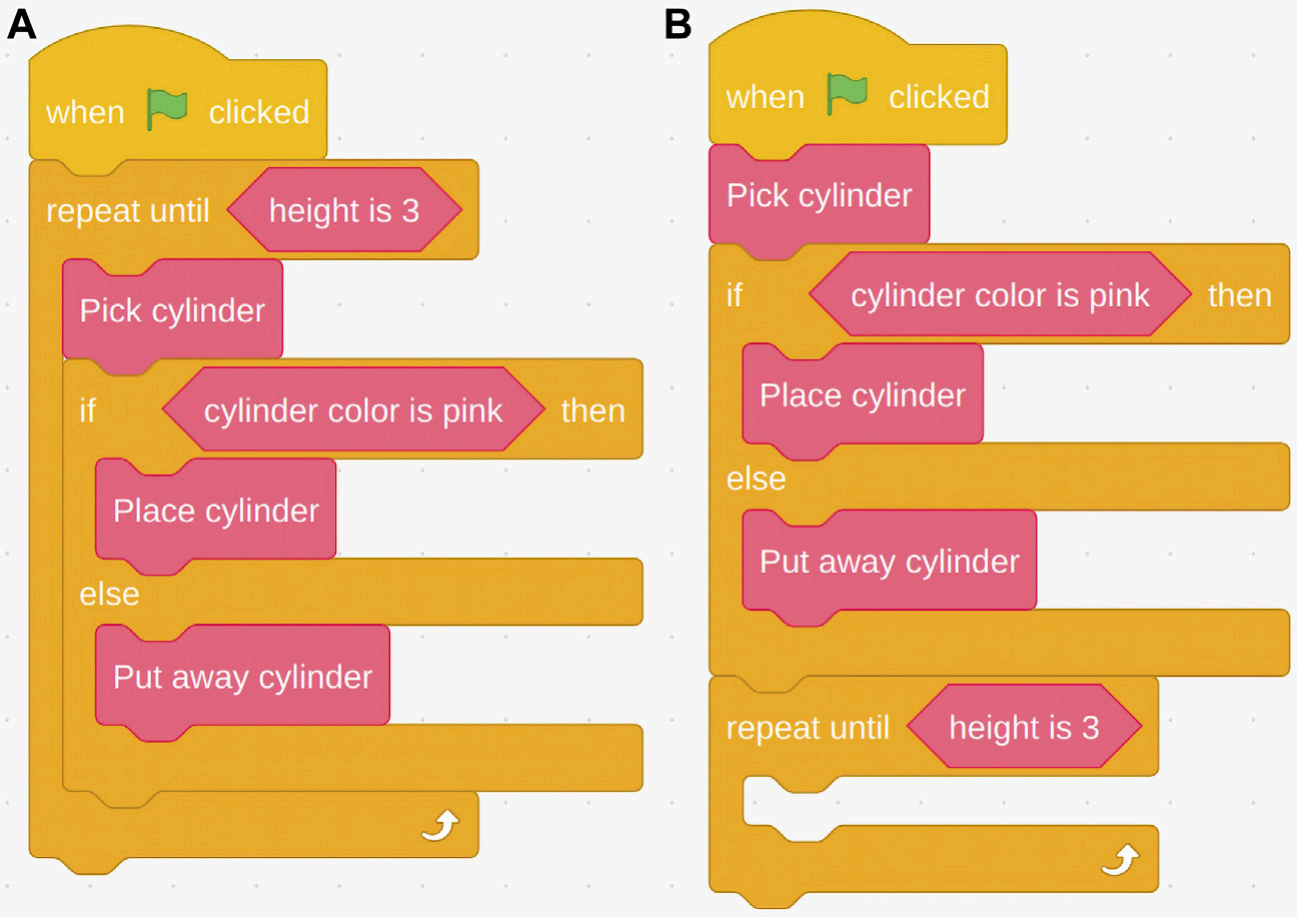
Pick-and-Place task: Correct program **(A)** and the loop incorrectly placed at end of program rather than around the “if-else” conditional **(B)**.

Table 2 reports the distribution of classified mistakes. The most common programming mistake was to place the loop at either the end of the program or at the beginning, without enveloping the rest of the blocks (see Figure 8). This problem started to occur in the loop subtask. 24% of the open answers were coded as issues with Block functionality. Participants explaining their problems said “(…) My thought was more like a timeline, so I put the block ‘Repeat Until Height is 3’ at the end expecting to function when the robot movement was done,” (loop subtask) and “I wasn’t exactly sure how to nest commands, the only thing I had to think about was where to put the repeat command (…)” (loop subtask). Another recurring mistake in the loop and variables subtasks was placing the loop inside the conditional statement, causing it to only execute if the conditional statement was true and only for the blocks inside that loop.

**TABLE 2:** Error classification in the pick-and-place task (improper use, IU; no use, NU) per programming statement for the conditional (C), loop (L), and variables (V) subtask.

Subtask	If-else		Loop		Variables	
	IU	NU	IU	NU	IU	NU
C	4 (18%)	0 (0%)	N/A	N/A	N/A	N/A
L	5 (21%)	0 (0%)	10 (42%)	6 (25%)	N/A	N/A
V	9 (36%)	2 (8%)	11 (44%)	3 (12%)	12 (48%)	1 (4%)

### 4.3 Summary of Findings

To summarize the results presented, we found that a third of the participants in the navigation task could successfully program the robot. People were significantly less likely to successfully complete the task in the conditional subtask as compared to the blocks subtask. More than half of the participants in the pick-and-place task could successfully create a simple robot program; people were significantly less likely to successfully complete the task in the loop subtask as compared to the conditional subtask. For both tasks, the task duration increased when more programming concepts were introduced. For the navigation task, the usability decreased between the blocks and conditional subtask and between the loop and variables subtask. For the navigation task, task load was significantly higher for the variables subtask as compared to the loop subtask; there was no significant difference for task load in the pick-and-place task. Inspecting the created programs and open answers, the most common mistakes were the order of blocks in the navigation task and the use of the loop block in the pick-and-place task.

## 5 Discussion and Future Work

This section discusses our findings on task success and common challenges as reported by non-experts during the robot programming task, and we discuss avenues for future work including the transfer from a simulation environment to integration with a real robot and quality control of the crowdworkers’ input.

### 5.1 Task Success

A higher percentage of workers succeeded in the pick-and-place task than in the navigation task, which may have been due to the non-deterministic nature of the cause for robot failure in the latter. People had difficulties handling the cat-event with basic switch logic; many participants placed a “move to hallway” command before the conditional statement to check if the cat blocked the robot’s way. Participants could re-try and adapt their program, but did not create the program successfully. Looking at people’s open answers, this difficulty may have been because the procedural if-statement may not align with their mental representation of the task at hand. It is reasonable that participants expected that the robot would start moving towards the hallway, and while moving, would execute the conditional statement in parallel rather than sequentially. It is important to note that participants could re-try, hence, they could observe how/when the robot executed its actions. According to the cognitive dimensions framework, it is crucial that there is a cognitive fit between the mental representation and language representation (Green and Petre, 1996). Especially because this difficulty was not a common challenge in the pick-and-place task, this suggests that participants may have had different mental representations of the two tasks.

### 5.2 Common Challenges

Interestingly, in the pick-and-place task, people experienced difficulties with the placement of the loop block, which was reflected in their open answers: almost a quarter of participants in the pick-and-place task mentioned issues related to how the blocks worked. The difference between the two tasks in terms of the loop statement is that in the navigation task only one action should be placed in the loop block, while in the pick-and-place task, a sequence of actions should be encapsulated in the loop block. It may have been unclear to participants that multiple blocks could be placed inside the loop block. Difficulties with proper programming block placement can most likely be solved by showing people a simple example and ensuring that workers watch the instruction video. In this study, participants started with a blank slate, starting with the initial robot program for which they can specify an additional rule could be a viable option as well. Another direction of important future work is the investigation of crowd workers who repeatedly complete robot programming tasks over a longer period of time. This may provide a more stable quality of the input we receive, but also create an interesting opportunity for the workers to become more familiar with robot programming.

### 5.3 Training Time

The use of non-experts for robot programming is first and foremost that the quick and easy access to a large pool of robot teachers. It is undesirable if people need to undergo extensive training before they are able to create simple rules and programs for robots. In other words, we envision a pipeline in which workers can quickly obtain the minimal skills to perform the task provided that their attempt is serious, so we wanted to keep the instructions to a minimum. Although non-significant, results suggested a potential increase in success rate in both the conditional (20.7–33.3%) and the loop subtask (10.5–28.6%) after adding only 30 s of video-based instructions explaining the functionality and use of a loop statement to a 1-min instruction video. Variable use, which has a different functionality than conditionals and loops, remained susceptible to errors (7.7% subtask success). This finding motivates the suggestion mentioned above, to have a smaller group of workers repeatedly provide input to a robot. This way, we could ask them to invest some time familiarizing themselves with the interface as they would be using it over a longer period of time. One specific approach that we want to study in the future is for non-experts to provide rules in natural language. For example, in case the robot is baking a cake and runs out of eggs on the counter (exception), a rule to enable quick robot learning: If there are no more eggs on the counter → Fetch eggs from the fridge. Then, more experienced crowd workers could translate this to a simple robot program. Leveraging collaboration between workers is one major advantage of crowdsourcing, which we have not yet explored in this current work.

### 5.4 Integration With a Real Robot

We conducted an initial study if/how well non-experts could create simple programs that specify additional rules for robots. This paper offers a first exploration, but does not offer a complete solution to non-expert robot programming using crowdsourcing. We explored the suitability of non-expert robot programming using an off-the-shelf visual programming interface that has proven intuitive for non-programmers on two typical robot programming tasks. Results provide initial evidence that non-experts can successfully create small robot programs using crowdsourcing and enjoy performing such tasks. However, this initial work focused on a simulation environment and hence, it is important to discuss the gap between this work and this approach on a real robot.

In addition to the two tasks presented in this work, we envision scenarios like a mobile robot in a nursing home assisting human workers to check whether all residents have left their rooms right before meal time or a mobile robot that guides people to their gate at the airport and should consider taking a longer, but more suitable route (e.g., without stairs) in special cases, e.g., when guiding elderly or visually impaired people. In our use case, the crowd is leveraged to provide additional rules to the robot behavior as implemented initially by experts. These rules will help the robot handle new situations that require adaptations of existing actions and behaviors, such as those that require a change in the sequence in which actions are performed.

This work is only the first step towards gathering collective intelligence for robots using crowdsourcing. We envision a pipeline that integrates a crowdsourcing platform with an autonomous robot deployed in the real world. Similar to Leite et al. (2016), the robot will continuously collect additional rules from crowd workers using semi-situated learning, a robot-human pipeline in which the robot autonomously decides when to request help from the crowd. Ideally, when the robot fails to accomplish a task, it can query workers for help to gradually learn adequate (social) behaviors and become increasingly autonomous. This method will not prevent the robot from failure, it instead focuses on failure cases that have not been anticipated at development. Such failures will inevitably happen at one point during development in the real world. At such moments, we need a human teacher to help the robot recover from its failure. Hiring an expert for each of these failures is expensive, hence, non-expert input provides an interesting and viable alternative for “common sense” failure cases in which non-experts can provide the additional rules the robot needs. Particularly, if the teacher can help the robot remotely, this would greatly improve teacher availability. Crowd workers would get partial information about the environment and the robot’s problem and create programs that allow the robot to achieve its goal. This requires us to consider some additional challenges. In this initial work, we assumed aspects such as sensing and world modeling as given, however, these need to be integrated and tests need to be done with crowd workers to investigate what information they need to provide meaningful and effective input to the robotic system.

Moreover, as robots will encounter new events for which they do not have detection yet, it is interesting to further explore to what extent workers can provide relevant information for detection and handling of those new events. We also must determine which parts of the pipeline are suitable for non-expert crowd workers to perform and which require more programming expertise. For example, program correction, debugging, and reward shaping to facilitate robot learning and sequential decision making are clear avenues for future work. More concretely, we are interested in using this non-expert robot programming pipeline in the following way: oftentimes, the rules non-experts can provide to the robot can be formulated in natural language (e.g., If *X*, then do *Y*, or Never do *X*), and can quickly be provided by crowd workers remotely. Such rules can be either automatically translated into constraints on the robot behavior, or we can let experienced non-experts (semi-experts) translate these rules into robot programs. Alternatively, program correction, for example, correcting synthesized programs by injecting additional rules into them, is an interesting alternative to explore. For both tasks, task duration, and load seemed to increase and usability seemed to decrease with the number of programming statements. Real-world tasks will likely involve a large number of programming statements and we must further study how to best distribute the work to crowd workers according to these factors.

In this study, participants could test their programs an unlimited number of times. Unlimited testing may not be desirable—or feasible in terms of time and cost—with a real robot. Alternative ways to provide adequate feedback to workers on program success need to be explored.

### 5.5 Quality of Crowd Input

We had to exclude 65 participants, of which a portion provided non-serious attempts at the task. Using crowd workers to monitor robots and create simple robot programs shifts the responsibility from a small group of experts to a large pool of non-experts, making data quality checks of utmost importance. For this, we need to explore ways to define robot task success for situations that the robot has not encountered before. When is crowd input “good?” When is the situation handled “successfully?” Can we define a measure of evaluation which allows the robotic system to autonomously evaluate its performance? How can we avoid deadlocks and too restricting constraints? It would be interesting to study to what extent non-experts can perform quality assurance. For example, an autonomous robot deployed in the real world may gather and store information throughout the day about the situations in which it fails and request input from workers during off-time [similar to Leite et al. (2016)], allowing for quality checks between obtaining and executing a new behavior. Then, if we can assure the quality of the constraints/rules from non-experts, and if these rules are provided the form as described in this work, we can define them in terms of logic (e.g., temporal logic), this would mean that we can formally verify the robot’s behavior and it does not contain deadlocks, and adheres to the set of constraints. Prior work has investigated whether people could create human-robot interactions when given a set of constraints (Porfirio et al., 2018), and our approach would complement this by letting people define additional constraints that were impossible to anticipate at design time. Equally important is to design such a pipeline in a responsible way, which means that the pipeline design “seeks the most benefit to society and the least harm to the environment” (p.9 Winfield et al., 2020). If, while under deployment, a robot shares information about its environment, the task, and the interactions it has, then the people who work alongside or with this robot need to be made aware of and consent to that.

## 6 Conclusion

A timely and persistent challenge in deploying robots in the real world is their versatility. To date, robot programming remains primarily a task done by engineers and expert programmers, which can be costly and inconvenient. We posit that robot programming includes a set of common knowledge tasks and propose to scale robot programming to the crowd. We presented initial findings on collecting simple robot programs as created by non-experts using crowdsourcing.

Our findings provide initial support for this approach, demonstrating that non-experts can successfully create simple programs after 1-min instructions. Overall, workers mentioned enjoying the task—even for tasks with an increased number of programming statements—and grasped programming statements (e.g., conditional, loop) that were explained or were of a similar nature as the statements explained in a one- or 1.5-min instruction video, and their task success seemed to increase with minimal additional instructions.

Clear avenues for future work include a study of roles for crowd workers (e.g., debugging, program correction), defining “success” measures to allow for autonomous queries to the crowd, and a longitudinal investigation in which crowd workers repeatedly provide input to the robot that it autonomously adds to its behavior repertoire over time. All in all, this initial exploration provides important insights into if/how we can use non-experts to help develop more versatile and robust robots that can operate over longer periods of time.

## Data Availability

The raw data supporting the conclusion of this article will be made available by the authors, without undue reservation.
